# Metabolic Changes and Serum Ghrelin Level in Patients with Psoriasis

**DOI:** 10.1155/2014/175693

**Published:** 2014-12-18

**Authors:** Haydar Ucak, Betul Demir, Demet Cicek, Ilker Erden, Suleyman Aydin, Selma Bakar Dertlioglu, Mustafa Arica

**Affiliations:** ^1^Department of Dermatology, Faculty of Medicine, Dicle University, 21070 Diyarbakir, Turkey; ^2^Department of Dermatology, Faculty of Medicine, Firat University, Elazig, Turkey; ^3^Department of Dermatology, Elazig Training and Research Hospital, Elazig, Turkey; ^4^Department of Biochemistry, Faculty of Medicine, Firat University, Elazig, Turkey

## Abstract

*Background*. Serum ghrelin levels may be related to metabolic and clinical changes in patients with psoriasis.* Objective*. This study was performed to determine the possible effects of serum ghrelin in patients with psoriasis.* Methods*. The study population consisted of 25 patients with plaque psoriasis. The patients were questioned with regard to age, gender, age of onset, duration of disease, height, weight, and body mass index (BMI). In addition, fasting blood sugar, triglyceride, cholesterol levels, insulin, and ghrelin levels were measured.* Results*. The mean serum ghrelin level was 45.41 ± 22.41 in the psoriasis group and 29.92 ± 14.65 in the healthy control group. Serum ghrelin level was significantly higher in the psoriasis group compared with the controls (*P* = 0.01). The mean ghrelin level in patients with a lower PASI score was significantly higher than in those with a higher PASI score (*P* = 0.02).* Conclusion*. The present study was performed to determine the effects of ghrelin in psoriasis patients. We found a negative correlation between severity of psoriasis and ghrelin level. Larger and especially experimental studies focusing on correlation of immune system-ghrelin levels and severity of psoriasis may be valuable to clarify the etiopathogenesis of the disease.

## 1. Introduction

Psoriasis is a chronic inflammatory disease that affects approximately 1–3% of the general population [1]. In addition, psoriasis is characterized by local and systemic increases in levels of proinflammatory cytokines, such as interleukin-6 (IL-6) and tumor necrosis factor-alpha (TNF-*α*) [2]. Proinflammatory cytokines in chronic inflammation lead to atherogenesis and peripheral insulin resistance, which in turn cause hypertension and type II diabetes mellitus (DM) [3, 4]. In addition, recent studies have indicated a relationship between psoriasis and metabolic syndrome [5, 6].

Ghrelin is a 28-amino acid peptide hormone secreted mainly by the mucosa of the stomach [7]. Ghrelin, thought to be a stimulator of growth hormone (GH) secretion [7] and food intake [8], also shows potent inhibitory effects on proinflammatory mediators via its effect on T cells and monocytes [9].

Previous studies have focused on the relationship between metabolic syndrome and psoriasis. The present study was performed to determine the effects of ghrelin on metabolic changes and the correlation between ghrelin level and disease severity in patients with psoriasis.

## 2. Patients and Methods

The study population consisted of 25 patients with chronic plaque psoriasis patients (11 males and 14 females) all of whom had been referred to the Department of Dermatology of Elazig Education and Research Hospital and Department of Dermatology of Firat University Hospital. Twenty-five healthy control subjects were also enrolled in the present study. All psoriasis patients had symptoms for at least 6 months and had not received any systemic or local antipsoriatic treatment for the last 4 weeks.

### 2.1. Exclusion Criteria

1. Patients with pustular psoriasis, erythroderma, and psoriatic arthritis,
2. Systemic diseases (diabetes and hypertension),
3. Age < 18 years,
4. Pregnancy,
5. Acute or chronic infection,
6. Acute or chronic neurological disorders,
7. Polycystic ovary syndrome or amenorrhea,
8. Hyperthyroidism or hypothyroidism.

### 2.2. Study Plan

The patients enrolled in the present study were questioned regarding age, gender, age of onset, duration of disease, history of smoking and alcohol use, height, weight, body mass index (BMI), and waist circumference. In addition, fasting blood sugar, triglyceride, low-density lipoprotein (LDL), very-low-density lipoprotein (VLDL), high-density lipoprotein (HDL), total cholesterol, HbA1c, insulin, C-peptide levels, thyroid stimulating hormone (TSH), T3, T4, and ghrelin levels were measured. Ghrelin levels may be affected by many metabolic factors, so patients and controls had similar BMI to decrease the different metabolic factors in patients with psoriasis.

The extent and severity of lesions were determined using the psoriasis area severity index (PASI) scoring system [10], body surface area (BSA) [11], and quality of life (QOL) was evaluated by calculating the Dermatology Life Quality Index score, which was tested previously for validity and reliability in Turkish by Öztürkcan et al. [12].

BMI was calculated as weight/height (kg/m^2^) and metabolic syndrome was diagnosed in the presence of central obesity in addition to two or more criteria of the international Diabetes Foundation: waist circumference 94 cm in males or ≥80 cm in females; hypertriglyceridemia ≥150 mg/dL; HDL <40 mg/dL in males or <50 mg/dL in females; blood pressure ≥130/85 mmHg; fasting blood glucose ≥ 100 mg/dL [13]. In addition, insulin resistance was calculated according to the homeostasis model assessment of insulin resistance (HOMA-IR) formula: (0 min glucose mg/dL × 0 min insulin *μ*U/mL)/405. Cases with a HOMA-IR index of >3.2 were diagnosed as having insulin resistance [14].

The control group comprised age-, sex-, and BMI-matched individuals who did not have any systemic or neurological diseases and did not use drugs or alcohol.

### 2.3. Collection and Storage of Biological Samples

As ghrelin is a peptide hormone and can be broken down by proteases, aprotinin (500 Kallikrein units per mL) was added to plain biochemistry tubes before collection of blood samples from the participants to prevent proteolysis. Blood samples were collected at 09.00–10.00 in the morning after an overnight fast to avoid any effects associated with circadian rhythm. Samples (5 mL) were collected from each participant after fasting and centrifuged at 3000 ×g for 5 min. The sera obtained were transferred to Eppendorf tubes and frozen at −80°C until the day of analysis.

Serum ghrelin levels were studied using a Human ghrelin kit (Cat. No. A05106; SPI-Bio, Montigny le Bretonneux, France) by the enzyme-linked immunosorbent assay (ELISA) method according to the manufacturer's instructions. According to the kit's supplier, the intra- and interassay coefficients of variation (CV) for this kit are <7% and <8.1%, respectively.

### 2.4. Statistical Analysis

SPSS version 12.0 (SPSS, Chicago, IL) was used for statistical analyses. The data obtained in the study are expressed as the means ± SD. The independent samples *t*-test and Mann-Whitney *U* test were used to compare groups. In all analyses, *P* < 0.05 was taken to indicate statistical significance.

## 3. Results

The study population consisted of 25 patients with psoriasis who presented at the Dermatology Polyclinics of Elazig Training and Research Hospital and Firat University Medical School Hospital. Twenty-five healthy volunteers were also included in the control group. The mean ages of the participants were 32.24 ± 7.54 years for the patients with psoriasis and 31.40 ± 6.77 years for the control subjects. The female-to-male ratio (F/M) was 14/11 in all groups. There were no significant differences between the groups in terms of mean age or gender (*P* > 0.05). Similarly, there was no significant difference in BMI between the psoriasis and control groups (*P* > 0.05). The demographic characteristics and clinical findings of the two groups are presented in Table 1.

The mean disease duration in psoriasis patients was 11.84 ± 7.79 years. In addition, nail involvement was observed in 5 patients (20.0%), genital involvement in 3 patients (12.0%), and scalp involvement in 20 patients (80.0%). Disease involvement score, PASI scores, and DLQI scores are presented in Table 2.

The relationships between involvement/severity of psoriasis and QOL were examined, and higher PASI score was shown to be associated with poorer QOL—the QOL scores were 6.00 ± 3.42, 6.37 ± 2.61, and 7.50 ± 4.78 in patients with low, moderate, and high PASI scores, respectively. However, the differences among these groups were not statistically significant (*P* > 0.05).

**Figure 1:**
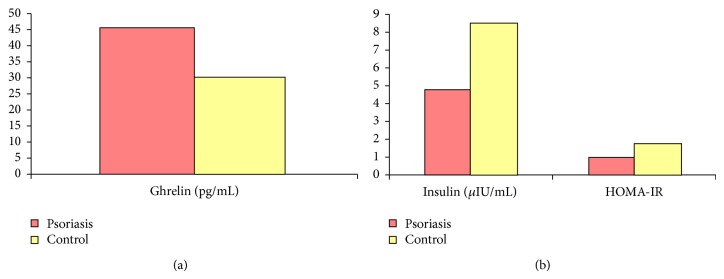
Serum ghrelin, insulin, and HOMA-IR levels.

The mean serum ghrelin level was 45.41 ± 22.41 in the psoriasis group and 29.92 ± 14.65 in the healthy control group, and this difference was significant (*P* = 0.01) (Table 3, Figure 1).

Mean serum insulin level and HOMA-IR index in the psoriasis group (4.79 ± 3.46 and 0.99 ± 0.70, resp.) were significantly lower than those in the healthy controls (8.56 ± 4.33 and 1.80 ± 0.97, resp.) (*P* = 0.002 and *P* = 0.001, resp.) (Table 3, Figure 1). Insulin resistance was observed in two patients in the psoriasis group (8.0%) and four subjects in the control group (16.0%). As the number of patients with insulin resistance was low, it was not possible to statistically compare the ghrelin levels of the subjects with and without insulin resistance. In addition, insulin level showed positive correlations with both C-peptide and HOMA-IR levels (*r* = 0.64, *P* < 0.001; *r* = 0.98, *P* < 0.001, resp.).

Metabolic syndrome was found in 10 (40.0%) psoriasis patients and 9 (36.0%) controls. The mean serum ghrelin level was higher in psoriasis patients with than in those without metabolic syndrome but the difference was not significant (54.21 ± 23.02 and 39.55 ± 20.69, resp.; *P* > 0.05). In addition, mean age and BSA in psoriasis patients with metabolic syndrome (36.20 ± 8.65, 27.62 ± 19.98, resp.) were significantly higher than those in patients without metabolic syndrome (29.60 ± 5.55, 12.78 ± 10.74, resp.) (*P* = 0.02, *P* = 0.02, resp.). In our study 10 psoriasis patients had MetS. 3 patients (30%) had mild psoriasis, 4 patients (40%) has moderate psoriasis, and 3 patients (30%) had severe psoriasis. MetS ratio in patients who had low PASI score was not higher than the other groups.

There were no significant differences in PASI, DLQI, or disease duration between the psoriasis patients with and without metabolic syndrome (all *P* > 0.05). The mean serum ghrelin levels were not significantly different between female and male patients (48.04 ± 23.99 and 42.07 ± 20.85, resp.; *P* > 0.05).

**Figure 2:**
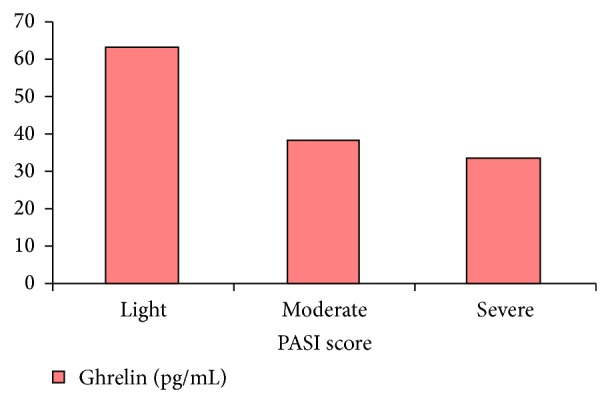
Serum ghrelin levels according to PASI score.

The relation between severity of psoriasis and ghrelin level was examined, and the results indicated that higher PASI score was associated with lower ghrelin level. The ghrelin levels were 62.66 ± 25.00, 37.89 ± 11.35, and 33.53 ± 16.43 in patients with low, moderate, and high PASI scores, respectively. The mean ghrelin level in patients with a lower PASI score was significantly higher than in those with a higher PASI score (*P* = 0.02) (Figure 2).

There was a positive correlation between BMI and DLQI (*r* = 0.46, *P* < 0.05). Therefore, higher BMI negatively affects QOL in patients with psoriasis. Interestingly, there was a negative correlation between PASI and serum ghrelin level (*r* = 0.49, *P* < 0.05).

## 4. Discussion

Metabolic syndrome affects approximately 15–25% of the general population [15, 16]. In addition, recent studies suggested that psoriasis patients may have an increased prevalence of metabolic syndrome. The reported prevalence of metabolic syndrome among patients with psoriasis ranges from 14% to 40% [17]. In the present study, metabolic syndrome was seen in 40% of psoriasis patients, which is consistent with the incidences reported in the literature.

The mechanism underlying the etiology of metabolic syndrome in patients with psoriasis is not fully understood. Some studies have suggested that psoriasis predisposes patients to the development of obesity or hypertension related to stress and reduced physical activity [18]. Therefore, insulin resistance and abdominal obesity are considered to play important roles in the pathogenesis of metabolic syndrome [19, 20]. In addition, Sommer et al. [21] found significant associations between psoriasis and type II DM, hypertension, hyperlipidemia, and coronary artery disease in a study of 581 patients. In the present study, we found no differences between psoriasis patients and controls with regard to fasting blood glucose, triglyceride, cholesterol, HDL, LDL, or VLDL levels. In addition, Naldi et al. [22] reported an association between BMI and psoriasis, and found a higher risk of psoriasis in the obese population. However, we found no differences in BMI or waist circumference among groups in the present study. These discrepancies between the present and previous studies may have been related to the similarity in BMI between patients and controls in our study.

There is controversy in the literature about the relationship between metabolic syndrome and severity of psoriasis. Sommer et al. [21] reported a positive correlation between metabolic syndrome and severity of the disease, while Gisondi et al. [23] reported no such correlation. We did not find a positive correlation between PASI and metabolic syndrome in the present study, but BSA was positively correlated with metabolic syndrome in our subjects.

A few studies have indicated relationships between elevated levels of inflammatory mediators, such as IL-6, TNF-*α*, and C-reactive protein levels, and metabolic syndrome [24]. In addition, psoriasis is a chronic inflammatory skin disorder characterized by a variety of immunological and inflammatory changes. Therefore, the link between psoriasis and metabolic syndrome may be related to the effects of chronic inflammatory changes and the secretion of proinflammatory cytokines [25].

Ghrelin is produced predominantly by the stomach, but is also expressed in other tissues, such as the hypothalamus, pituitary gland, intestine, pancreas, kidney, placenta, testes, ovary, and lymphocytes [26]. There has been only one previous study regarding ghrelin levels in patients with psoriasis. Özdemir et al. [27] reported that the ghrelin level was higher in psoriasis patients than in controls, but the difference was not statistically significant. In the present study, ghrelin levels were significantly higher in psoriasis patients compared to controls. In addition, the levels of insulin and HOMA-IR were significantly lower in patients than in controls. These observations may be related to possible effects of ghrelin on insulin homeostasis. In addition, we found higher levels of ghrelin in psoriasis patients with than without metabolic syndrome, although the difference was not significant. This finding may have been related to the presence of many factors that affect ghrelin levels in psoriasis and metabolic syndrome.

Özdemir et al. [27] reported a negative correlation between ghrelin level and the severity of psoriasis. Interestingly, we also found a negative correlation between ghrelin level and PASI score. Some studies indicated that ghrelin has potent inhibitory effects on the mRNA and protein expression levels of proinflammatory cytokines, such as IL-6 and TNF-*α* [28], which are important in the pathogenesis of psoriasis. In addition, Xia et al. [29] reported that ghrelin inhibits proliferation of anti-CD3-activated murine T cells and nonspecifically inhibits both Th1 (IL-1 and INF-*γ*) and Th2 (IL-4 and IL-10) cytokines. Arican et al. [30] reported positive correlations between the severity of psoriasis and serum TNF-*α*, IL-6, and IL-8 levels. This correlation may have been responsible for the negative correlation between PASI score and ghrelin levels observed in the present study.

This study had some limitations, such as the small number of patients. In addition, serum levels of ghrelin may be affected by various endogenous and exogenous factors, such as diet and decreased physical activity, which could not be compared in our study. Moreover, we could not study immunological parameters associated with metabolic syndrome and psoriasis that may have been affected by ghrelin.

## 5. Conclusions

The pathogenesis of psoriasis and presence of metabolic syndrome in psoriasis are not fully understood. Recent studies discussed the relations between serum ghrelin levels and both metabolic syndrome and psoriasis. The present study was performed to determine the effects of ghrelin in psoriasis patients. We found a negative correlation between severity of psoriasis and ghrelin level. In addition, we found correlations between ghrelin levels and some metabolic changes. Larger and especially experimental studies focusing on the correlations between the immune system, ghrelin levels, and severity of psoriasis may be valuable to clarify the etiopathogenesis of the disease and to improve treatment alternatives in patients with psoriasis.

## Figures and Tables

**Table 1:** Demographical and clinical findings of groups.

	*Psoriasis vulgaris *	Control	*P*
*n*	25	25	
Gender (F/M)	14/11	14/11	*P* > 0.05
Age^*^ (year)	32.24 ± 7.54	31.40 ± 6.77	*P* > 0.05
BMI^*^ (kg/m^2^)	24.96 ± 2.53	23.80 ± 1.50	*P* > 0.05
BMI score^*^	2.48 ± 0.65	2.28 ± 0.45	*P* > 0.05
Waist circumference^*^ (cm)	86.84 ± 10.22	83.72 ± 8.48	*P* > 0.05

^*^(Mean ± SD).

**Table 2:** Body involvement percentage and PASI and DLQI values of patients with psoriasis.

	Patients with psoriasis		*n* (%)
PASI^*^		5.59 ± 4.38	

PASI score^*^		1.96 ± 0.84	

PASI score dissociation	Mild		9 (36.0)
	Moderate		8 (32.0)
	Severe		8 (32.0)

Body involvement percentage^*^		18.72 ± 16.49	

DLQI^*^		6.60 ± 3.60	

DLQI score^*^		1.72 ± 0.73	

DLQI score dissociation	No efficacy		1 (4.0)
	Low efficacy		8 (32.0)
	Moderate efficacy		13 (52.0)
	Major efficacy		3 (12.0)
	Colossal efficacy		0 (0)

^*^(Mean ± SD).

**Table 3:** Laboratory findings of patient and control groups.

Parameter	*Psoriasis vulgaris *	Control	*P*
Glucose^*^ (mg/dL)	85.80 ± 11.88	84.96 ± 10.94	*P* > 0.05
Triglycerides^*^ (mg/dL)	129.52 ± 71.80	125.08 ± 90.56	*P* > 0.05
LDL-cholesterol^*^ (mg/dL)	112.75 ± 33.77	103.02 ± 34.88	*P* > 0.05
HDL-cholesterol^*^ (mg/dL)	44.48 ± 13.47	47.48 ± 13.26	*P* > 0.05
Total cholesterol^*^ (mg/dL)	182.64 ± 37.29	179.92 ± 26.87	*P* > 0.05
Insulin^*^ (*µ*IU/mL)	4.79 ± 3.46	8.56 ± 4.33	**P** = 0.002
C-peptide^*^ (ng/mL)	1.95 ± 0.73	1.88 ± 0.66	*P* > 0.05
HOMA-IR values^*^	0.99 ± 0.70	1.80 ± 0.97	**P** = 0.001
Ghrelin^*^ (pg/mL)	45.41 ± 22.41	29.92 ± 14.65	**P** = 0.01

^*^(Mean ± SD).
